# c-Jun N-Terminal Kinase as a Therapeutic Target in Experimental Autoimmune Encephalomyelitis

**DOI:** 10.3390/cells9102154

**Published:** 2020-09-23

**Authors:** Maud Bagnoud, Myriam Briner, Jana Remlinger, Ivo Meli, Sara Schuetz, Maximilian Pistor, Anke Salmen, Andrew Chan, Robert Hoepner

**Affiliations:** 1 Department of Neurology, Inselspital, Bern University Hospital, University of Bern, 3010 Bern, Switzerland; myriam.briner@insel.ch (M.B.); jana.remlinger@insel.ch (J.R.); ivo.meli@extern.insel.ch (I.M.); sara.schuetz@bioinformatics.unibe.ch (S.S.); maximilian.pistor@insel.ch (M.P.); anke.salmen@insel.ch (A.S.); andrew.chan@insel.ch (A.C.); robert.hoepner@insel.ch (R.H.); 2 Department of Biomedical Research, University of Bern, 3010 Bern, Switzerland; 3 Graduate School for Cellular and Biomedical Sciences, University of Bern, 3010 Bern, Switzerland

**Keywords:** multiple sclerosis, immunotherapy, mitogen-activated protein kinases, MAPK, SP600125, neuroinflammation

## Abstract

c-Jun N-terminal kinase (JNK) is upregulated during multiple sclerosis relapses and at the peak of experimental autoimmune encephalomyelitis (EAE). We aim to investigate the effects of pharmacological pan-JNK inhibition on the course of myelin oligodendrocyte glycoprotein (MOG_35-55_) EAE disease using in vivo and in vitro experimental models. EAE was induced in female C57BL/6JRj wild type mice using MOG_35-55_. SP600125 (SP), a reversible adenosine triphosphate competitive pan-JNK inhibitor, was then given orally after disease onset. Positive correlation between SP plasma and brain concentration was observed. Nine, but not three, consecutive days of SP treatment led to a significant dose-dependent decrease of mean cumulative MOG_35-55_ EAE severity that was associated with increased mRNA expression of interferon gamma (INF-γ) and tumor necrosis factor alpha (TNF-α) in the spinal cord. On a histological level, reduced spinal cord immune cell-infiltration predominantly of CD3+ T cells as well as increased activity of Iba1+ cells were observed in treated animals. In addition, in vitro incubation of murine and human CD3+ T cells with SP resulted in reduced T cell apoptosis and proliferation. In conclusion, our study demonstrates that pharmacological pan-JNK inhibition might be a treatment strategy for autoimmune central nervous system demyelination.

## 1. Introduction

Multiple sclerosis (MS) is an inflammatory and degenerative disorder of the central nervous system (CNS) [1]. Mitogen-activated protein kinases (MAPKs) have been shown to be involved in the regulation of cytokine gene expression, immune cell differentiation, neuronal-cell-death pathways and regulation of astrocyte inflammatory genes [2,3]. Consequently, they might influence both pathomechanisms of MS; neuroinflammation and degeneration. More precisely, MAPKs are divided into three subfamilies: The c-Jun N-terminal kinases (JNKs), the extracellular signal-regulated kinases (ERKs) and the p38 mitogen-activated protein kinases (p38) [4]. Originally, JNKs were named stress-activated protein kinases (SAPKs) due to their ability to respond to different environmental stressors such as ultraviolet irradiation, osmotic shock or oxidative stress [5]. Later, it was demonstrated that cytokines, hormones or morphogenic factors were also able to activate the c-Jun N-terminal kinase (JNK) pathway [6]. In mammals, three *Jnk* genes have been described. Of these, *Jnk1* and *Jnk2* are ubiquitously expressed, whereas *Jnk3* is exclusively found in heart, brain and testis [7].

The JNK pathway has been proposed to be an important player in several neurological and autoimmune disorders [8,9], and particular in MS where upregulation of JNK activity has been observed in peripheral blood mononuclear cells (PBMCs) of relapsing MS patients compared to healthy volunteers [10]. Moreover, an increase of phosphorylated JNK (p-JNK) was found in the spinal cords of myelin basic protein (MBP) experimental autoimmune encephalomyelitis (EAE) Lewis rats during the acute phase of the disease [11]. Non-specific as well as specific inhibition of the JNK pathway has also demonstrated some beneficial effects in the myelin oligodendrocyte glycoprotein (MOG_35-55_) EAE. For instance, N-acetylcysteine amide (AD4), a copper chelator, attenuates MOG_35-55_ disease severity by scavenging reactive oxygen species, by reducing matrix metallopeptidases activation and by inhibiting p38 and JNK phosphorylation [12]. It has also been shown that Paeoniflorin, which is extracted from the roots of Paeonia lactiflora, ameliorates MOG_35-55_ EAE disease severity by decreasing Th17 cell differentiation and by inhibiting dendritic cell function via the suppression of the IKK/NFkB and JNK pathway [13]. In addition, preventive treatment with SP600125 (SP), a JNK inhibitor, reduces the MOG_35-55_ EAE disease severity in wild type (WT) mice [14]. However, the genetic knockout of *Jnk2* did not lead to differences in MOG_35-55_. EAE disease course compared to wild type C57BL/6JRj (C57BL/6) mice [15], whereas knockout of *Jnk1* moderately ameliorated EAE disease course [16]. 

A mouse model with a pan-JNK knockout as well as studies investigating the treatment effect of a pharmacological pan-JNK inhibition in a therapeutic setting are lacking. 

Therefore, our goal was to investigate, whether pharmacological inhibition of all JNK isoforms in a therapeutic setting may have the potential to ameliorate MOG_35-55_ EAE disease course. Given positive findings in vivo, we further aimed at subsequently translating the results to the human situation by performing in vitro studies in murine and human CD3+ T cells in parallel. 

## 2. Materials and Methods

Animal and human studies were approved by the local authorities (Office of Agriculture and Nature Bern, Switzerland: 101/16; Cantonal Ethic Committee Bern, Switzerland: 2017-00060). C57BL/6JRj (C57BL/6) WT mice were ordered from Janvier Labs (Le-Genest-Saint-Isle, France) and housed under conventional housing conditions at the in-house animal facility of the University of Bern.

### 2.1. In Vivo and In Vitro Treatments

JNK inhibition was performed using SP600125 (SP), a pan-JNK inhibitor (Seleck Chemicals, Houston, TX, USA). SP was chosen for our experiments as it is a non-toxic reversible adenosine triphosphate (ATP) competitive inhibitor of JNK with similar specificity especially for the immunological relevant JNK isoforms 1 and 2 [17]. SP concentration used for the in vitro experiments was set to 10 µM and for in vivo experiments to 15 and 30 mg/kg/day. 

### 2.2. Active Myelin Oligodendrocyte Glycoprotein (MOG_35-55_) Experimental Autoimmune Encephalomyelitis (MOG_35-55_ EAE)

Active MOG_35-55_ EAE was induced in 8 weeks old female C57BL/6 WT mice following our previously described MOG_35-55_ EAE induction protocol [18]. Briefly, animals were immunized by subcutaneous injection of 100 µg MOG_35-55_ peptide (Institute of Medical Immunology, Charité, Berlin, Germany) in phosphate-buffered saline solution (PBS; Thermo Fisher Scientific, Dreieich, Germany) emulsified in complete Freund’s adjuvant containing 100 µg mycobacterium tuberculosis (Difco, Detroit, MI, USA), followed by 200 ng pertussis toxin (Quadratech, Epsom, UK; intraperitoneal (i.p.) injection, days 0 and 2). Animals were scored daily in a blinded manner using a 10-point EAE scale [18]. In addition to EAE scoring, body weight, which is a well-established parameter of drug side effects [19], was measured daily and a clinical assessment of side effects based on body weight and observation of the animals’ behavior was performed. Treatment was initiated when animals had an EAE score ≥2. SP (15 or 30 mg/kg/day (Sigma-Aldrich, MO, USA), solvent: Dimethyl sulfoxide (DMSO, final DMSO (Sigma-Aldrich) concentration 10% in peanut oil (Migros, Zurich, Switzerland)) oral gavage; and control (10% DMSO in peanut oil, oral gavage) were given on three or nine consecutive days. 

### 2.3. Apoptosis Assay

CD3+ T cells were isolated from the spleen of C57BL/6 WT mice or from human PBMCs sampled from healthy donors by negative magnetic-activated cell sorting (MACS) selection (Pan-T cell isolation kit®, Miltenyi Biotec, NRW, Germany). CD3+ T cells were resuspended at 1mio/ml in T cell medium (RPMI 1640 medium, Life Technologies, CA, USA) containing 10% fetal bovine serum (FBS, Life Technologies), 1% penicillin/streptomycin (P/S, Thermo Fisher Scientific) and 1% L-glutamine (Life Technologies)). Cells were plated in a round bottom 96-well plate at a concentration of 100 000 cells/well. Murine CD3+ T cells were stimulated with concanavalin A (conA, 1.5 µg/mL, Sigma-Aldrich) and human CD3+ T cells with phytohemagglutinin (PHA, 2.5 µg/mL, Sigma-Aldrich). Both murine and human CD3+ T cells were treated with SP (10 µM, dissolved in DMSO, final DMSO concentration in the experiment 0.1%). Apoptosis (Annexin V/Propidium iodide, Becton Dickinson Bioscience, NJ, USA) was analyzed by flow cytometry after 24 h for murine and 72 h for human CD3+ T cells, respectively.

### 2.4. Proliferation Assay

Murine and human CD3+ T cells were isolated and cultivated as described in the subsection apoptosis assay. Murine cells were collected after 48 h and human cells after 72 h and proliferation was assessed by flow cytometry using carboxyfluorescein succinimidyl ester staining (CFSE, Thermo Fisher Scientific). 

### 2.5. RNA Isolation

**Figure A1:**
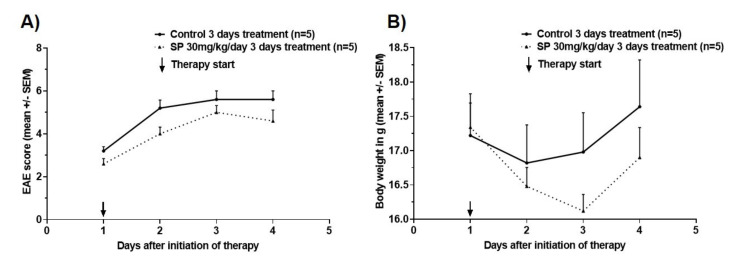
(**A**) clinical disease course of MOG_35-55_ EAE in C57BL/6JRj wild type mice treated for three consecutive days with control (DMSO in peanut oil) or SP600125 30 mg/kg/day. (**B**) body weight during course of MOG_35-55_ EAE in C57BL/6JRj wild type mice treated for three consecutive days with control (DMSO in peanut oil) or SP600125 30 mg/kg/day. Numbers of included animals are displayed in the graph. EAE score: 10 score system according to [18]. Abbreviations: EAE: Experimental autoimmune encephalomyelitis, SEM: Standard error of the mean and SP: SP600125. Statistic: Mann–Whitney Test: n.s.

**Figure A2:**
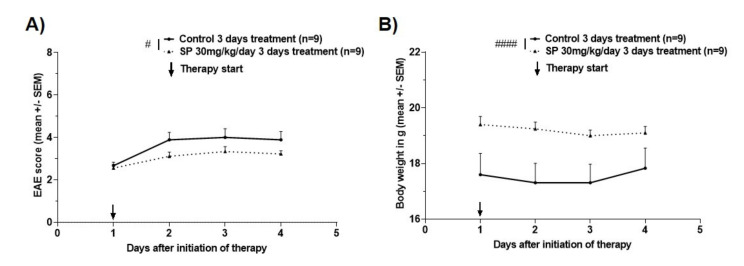
(**A**) clinical disease course of MOG_35-55_ EAE in C57BL/6JRj wild type mice treated for three consecutive days with control (DMSO in peanut oil) or SP600125 30 mg/kg/day. (**B**) body weight during course of MOG_35-55_ EAE in C57BL/6JRj wild type mice treated for three consecutive days with control (DMSO in peanut oil) or SP600125 30 mg/kg/day. Number of included animals are displayed in the graph. EAE score: 10 score system according to [16]. Abbreviations: EAE: Experimental autoimmune encephalomyelitis, SEM: Standard error of the mean and SP: SP600125. Statistic: Mann–Whitney Test: # < 0.05 and #### < 0.0001.

Splenic-derived CD3+ T cells, spinal cords and brains were isolated from control (CD3+ T cells and spinal cords: *n* = 5; brains: *n* = 9) or SP-treated (CD3+ T cells and spinal cords: *n* = 5; brains: *n* = 9; 30 mg/kg/day) mice at peak of MOG_35-55_ EAE disease corresponding to day 4 after treatment initiation (for disease course and body weight of the mice see Figure A1 and Figure A2). RNA was extracted following manufacturer’s instructions (RNeasy Kit, Qiagen, Hilden, Germany). Complementary DNA (cDNA) was prepared from 1 µg RNA mixed with Quanta qScript cDNA SuperMix (VWR International, Radnor, PA, USA). Reverse transcription was done using a thermal program of 5 min 25 °C, 30 min 42 °C and 5 min 85 °C. RNA was stored at −80 °C.

### 2.6. Quantitative Real-Time Reverse Transcription Polymerase Chain Reaction (qRT-PCR) and Analysis

Expression of interferon gamma (INF-γ), tumor necrosis factor alpha (TNF-α), interleukin-4 (IL-4), interleukin-10 (IL-10) and interleukin-17 (IL-17) genes was analyzed using qRT-PCR with primers from Thermo Fisher Scientific (INF-γ: Mm01168134_m1; TNF-α: Mm00443258_m1; IL-4: Mm445259_m1; IL-10: Mm01288386_m1; and IL-17: Mm00439618_m1). Transcripts were amplified using TaqMan qRT-PCR (TaqMan universal mastermix: KAPPA PROBE FAST Universal, Sigma-Aldrich, Louis, MO, USA). Experiments were run on an ABI7500 (Applied Biosystems, Foster City, CA, USA). Gene expression was normalized to housekeeping gene Rps18 coding for the Ribosomal Protein S18 (Mm02601777_g1, Thermo Fisher Scientific). The standard curve method of comparative quantification was used to analyze results [20].

### 2.7. Protein Analysis

Spinal cords and brains were isolated from control (*n* = 9) or SP-treated (*n* = 9, 30 mg/kg/day) mice at peak of MOG_35-55_ EAE disease corresponding to day 4 after treatment initiation (for disease course and body weight of the mice see Figure A2). Proteins were extracted following manufacturer’s instructions (Tissue Extraction Reagent I, Invitrogen, CA, USA). INF-γ and TNF-α protein concentrations were measured using commercially available ELISA kits (INF-γ: ab100690, Abcam, Cambridge, UK; TNF-α: 88-7324, Invitrogen).

### 2.8. Histology

Spinal cords were isolated from control (*n* = 9) or SP-treated (*n* = 9, 30 mg/kg/day) mice at peak of MOG_35-55_ EAE disease corresponding to day 4 after treatment initiation (for disease course and body weight of the mice see Figure A2), dehydrated and embedded in paraffin. Then, 5 µm thick tissue sections were stained with hematoxylin and eosin (H&E) or luxol fast blue/periodic acid Schiff. CD3+ and Mac3+ cells were detected via immunohistochemistry, whereas GFAP+ and Iba1+ cells were visualized after immunofluorescence staining (CD3 (primary antibody): Rat-α-human CD3, 1:100, AbD Serotec, Düsseldorf, Germany; Mac3 (primary antibody): Rat-α-mouse Mac3, 1:100; BD Pharmingen, Heidelberg, Germany; CD3 and Mac3 (secondary antibody): Biotinylated rabbit-α-rat IgG, 1:200, Vector Labs, CA, USA; GFAP (primary antibody): Chicken-α-GFAP, 1:1000, Abcam; GFAP (secondary antibody): Goat-α-chicken AF488, 1:1000, Thermo Fisher; and Iba1 (primary antibody): Rabbit-α-Iba1, 1:200, Wako, Osaka, Japan; Iba1 (secondary antibody): Goat-α-rabbit AF555, 1:1000, Thermo Fisher). Images were acquired with a slide scanner (Pannoramic 250 Flash III, 3DHISTECH, Budapest, Hungary). For each spinal cord segment (cervical, thoracic and lumbar), four different regions of interest (ROI) were examined except for demyelination where the complete white matter was analyzed. CD3+ and Mac3+ cell infiltrations were determined using CaseViewer (3DHISTECH). The percentage of white matter demyelination, GFAP and Iba1 fluorescence intensity as well as the roundness of Iba1+ cells were determined with ImageJ (National Institute of Mental Health, NIH, Bethesda, MD, USA).

### 2.9. Mass Spectrometry

The liquid chromatography–mass spectrometry (LC-MS) system with an autosampler was a Shimadzu LC-20A (Shimadzu, Japan) coupled with Applied Biosystem Sciex (MDS Sciex, Canada) API 5500 Tandem quadrapole mass spectrometer. The autosampler was SIL-HTC and the LC pump was LC-20AD, both from Shimadzu. The chromatographic integration was performed by Analyst software (version: 1.6.3; Applied Biosystems). Chromatographic separation was performed on a Phenomenex Kinetex C18 2.6u 2.1 by 50 mm analytical column and the mobile phase was 0.1% (*v*/*v*) formic acid in water and 0.1% (v/v) formic acid in acetonitrile with a gradient, injection volume of 6 µL and flow rate of 0.4 mL/min. Total analysis time of a single injection was 3 minutes and 30 seconds. Column oven temperature and autosampler temperature was set to 25 °C and 6 °C, respectively. 

The LC eluent was introduced via electrospray ionization using a Turbo IonSpray interface set at 600 °C to generate positive ions [M+H] +. Ionization was assisted with nebulizer and IonSpray gas (nitrogen) at 7 (arbitrary units) and 10 L/min, respectively. The IonSpray potential was maintained at 5 kV. During ms/ms analysis, the collision energies used ranged from 25 to 37 V and the scan speed was 10 Da/sec. Quantification was performed by multiple reaction monitoring of the protonated precursor ion and the related product ion for both test article and internal standard (IS, propranolol) using the IS method with a peak area ratio and a linear least-squares regression curve with weighting factor of 1/×2. The mass transitions used for test article and propranolol were *m*/*z* 221.0→165.0 and m/z 260.2→116.2, respectively.

Stock solutions of test article and propranolol were prepared in DMSO and further diluted in ethanol. Calibration standards of eight concentration levels were prepared freshly and spiked into drug-free plasma or brain homogenate with test article stock solutions to give concentrations of 0.00116, 0.00231, 0.00463, 0.0139, 0.0417, 0.125, 0.500 and 2.0 µM.

Brain samples (accurately weighed) were homogenized to a final dilution of 3× (*w*/*v*) in 50:50 methanol:water using ceramic beads. Vigorous shaking and rotation at high revolution (known as bead blasting) of the brain and diluent against the metal beads inside a capped sample tube disrupts the cellular structure and effects homogenization of individual samples with zero carryover in preparation, as the beads are only used once. Blank brain samples were treated in the same manner and used for the calibration curve matrix.

Plasma and brain homogenates were aliquoted and the test article was extracted via protein precipitation with 5 volumes of acetonitrile containing 20 nM propranolol as the internal standard vortexed for 5 minutes, followed by centrifugation at 4000 rpm/min on a cooling centrifuge for 10 minutes at 25 °C. The supernatant was withdrawn and diluted 1:1 with LC-MS grade water before injection on the LC-MS system. The limit of quantification was of 1.16 nM. 

### 2.10. Statistical Analysis

Data are expressed as mean +/− standard error of the mean (SEM). Comparison of data was performed using Kruskal–Wallis Test (KWT) or Mann–Whitney test (MWT), depending on the number of group comparisons, for independent and Wilcoxon signed rank sum test (WSRST) for dependent measurements. Association between SP serum and brain tissue concentration was analyzed using Pearson correlation coefficient. Gene expression and protein data were calculated as X-fold change of SP compared to control-treated animals and plotted as mean and 95%-confidence interval (95%-CI). In this analysis—as 1-fold represents no significant difference—a one-sample t-test (OSTT) was used with the test value of 1 to investigate gene regulation by SP. To determine if EAE score or cumulative SP dose influence body weight, a multivariate linear regression analysis was run. In this regression models, body weight at end of disease was used as dependent variable and EAE score, dose group of SP and an interaction term between the latter as independent variables. Data of the regression analysis is given as coefficient (coef) and 95%-CI. Significance was assumed for all tests if *p*-value was <0.05. 

## 3. Results

### 3.1. Plasma and Brain SP Concentrations of SP 30 mg/kg/day-Treated Acute MOG_35-55_ EAE-Diseased Mice Positively Correlate

**Figure 1:**
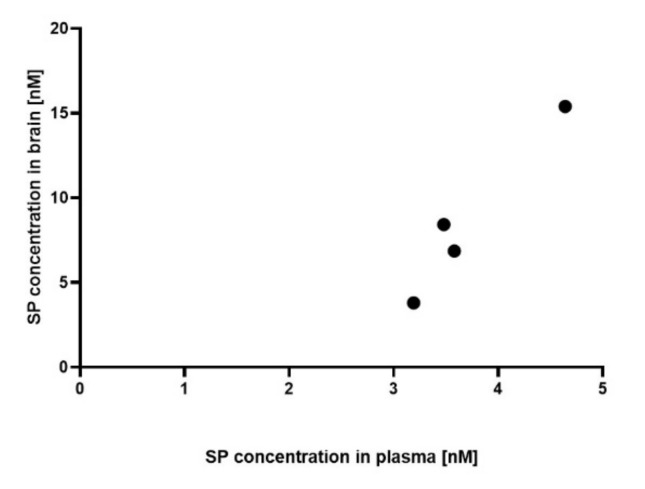
Association between plasma and brain SP600125 (SP) concentration. SP concentration was measured both in the plasma and in the brain of SP 30 mg/kg/day-treated acute myelin oligodendrocyte glycoprotein (MOG_35-55_) experimental autoimmune encephalomyelitis (EAE)-diseased mice (*n* = 4). *n* = 2 pairs had no detectable values in brain or plasma samples and were excluded. Mass spectrometry. Abbreviations: SP: SP600125. Statistic: Pearson r correlation, coefficient 0.97, 95% CI: 0.19-1.0 and *p* < 0.05.

Preliminary data using a small cohort demonstrated that SP was detectable in the plasma of 5/6 and in the brain of 4/6 SP 30 mg/kg/day-treated acute MOG_35-55_ EAE-diseased mice, whereas it was undetectable in all four control mice. In more details, the mean plasma SP concentration of SP 30 mg/kg/day-treated acute MOG_35-55_ EAE-diseased mice was of 3.35 nM (SEM 0.45, *n* = 5), whereas the mean SP brain concentration was of 8.62 nM (SEM 2.46, *n* = 4). Furthermore, positive correlation between SP plasma and brain concentration was noticed (Pearson r correlation, coefficient 0.97, 95% CI: 0.19–1.0, *p* < 0.05, Figure 1).

### 3.2. Effects of Pharmacological Pan-JNK Inhibition on MOG_35-55_ EAE Disease Course

**Figure 2:**
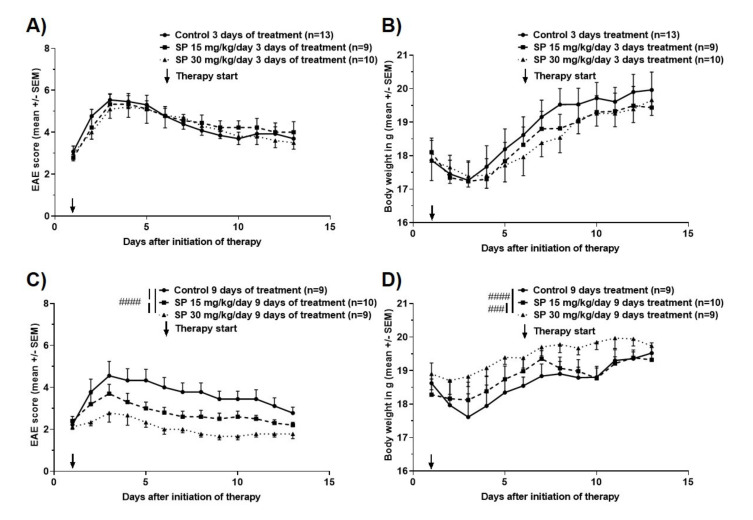
Clinical disease course of MOG_35-55_ EAE in C57BL/6JRj wild type mice treated for (**A**) three or (**C**) nine consecutive days with control (dimethyl sulfoxide (DMSO (in peanut oil), SP600125 15 or 30 mg/kg/day. Body weight during course of MOG_35-55_ EAE in C57BL/6JRj wild type mice treated for (**B**) three or (**D**) nine consecutive days with control (DMSO in peanut oil), SP600125 15 or 30 mg/kg/day. Numbers of included animals are displayed in the graph. EAE score: 10 score system according to [18]. Abbreviations: EAE: Experimental autoimmune encephalomyelitis, SEM: Standard error of the mean and SP: SP600125. Statistic: Kruskal–Wallis Test: ### < 0.001 and #### < 0.0001.

Two experimental setups were used to evaluate if pan-JNK inhibition is a potential strategy for treatment of acute demyelinating events: Drug administration over three consecutive days in analogy to treatment of MS relapses in the human setting versus drug administration over nine consecutive days imitating chronic treatment of autoimmune neuroinflammation. Short-term treatment over three consecutive days with either 15 or 30 mg/kg/day had no effect on the mean cumulative MOG_35-55_ EAE score (KWT: Each *p* > 0.05; Figure 2A) and on body weight (KWT: Each *p* > 0.05; Figure 2B). In contrast, administration over nine consecutive days led to a reduction of mean cumulative MOG_35-55_ EAE score in the SP 15 mg/kg/day group by 24.0% (KWT: *p* < 0.0001; Figure 2C) and in the 30 mg/kg/day group by 43.3% (KWT: *p* < 0.0001; Figure 2C) compared to control treatment. This reduced clinical severity was accompanied by higher body weights of the 15 and 30 mg/kg/day SP-treated animals (mean percentage of body weight changes compared to control: SP 15 mg/kg/day +0.9% (KWT: *p* > 0.05) and SP 30 mg/kg/day +4.3% (KWT: *p* < 0.0001); Figure 2D). 

In both experimental setups and treatment arms, no SP side effects on gross animal behavior were observed. Multivariate regression analysis demonstrated that weight loss was independent of treatment (3 days treatment: Coefficient −0.12 95%-CI -0.32–0.07, *p* = 0.21; 9 days treatment: Coefficient 0.02 95–%CI −0.11–0.16; *p* = 0.70), but predicted by MOG_35-55_ EAE score in the control as well as the SP groups (3 days treatment: Coefficient −4.7 95%-CI −5.2–−4.2, *p* < 0.001; 9 days treatment: Coefficient −8.1 95%–CI −9.1–−7.1; *p* < 0.001). 

### 3.3. JNK Inhibition Increases Spinal Cord INF-γ and TNF-α mRNA Expression during the Acute Phase of MOG_35-55_ EAE 

**Figure 3:**
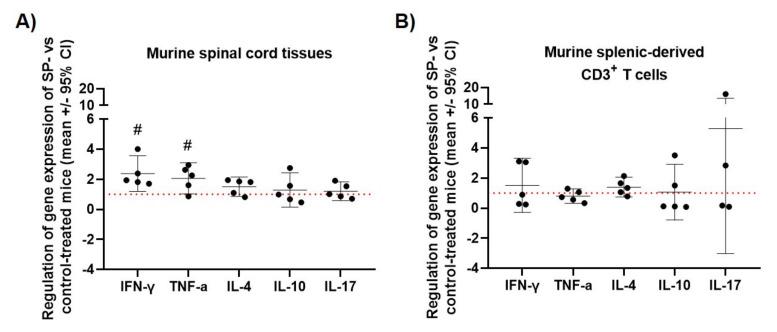
Effect of SP treatment on relative cytokine mRNA expression in (**A**) murine spinal cord of MOG_35-55_ EAE-diseased mice (acute phase) and in (**B**) murine splenic-derived CD3+ T cells of MOG_35-55_ EAE-diseased mice (acute phase). (**A**,**B**) X-fold difference in the relative expression of INF-γ, TNF-α, IL-4, IL-10 and IL-17 (normalized to Rps18) in SP 30 mg/kg/day-treated mice compared to control mice. Control (*n* = 5, in triplicates), SP 30 mg/kg/day (*n* = 5, in triplicates) and 3 days of treatment. qRT-PCR. Abbreviations: SP: SP600125, CI: Confidence of interval. Statistic: One-sample t-test # < 0.05.

**Figure A3:**
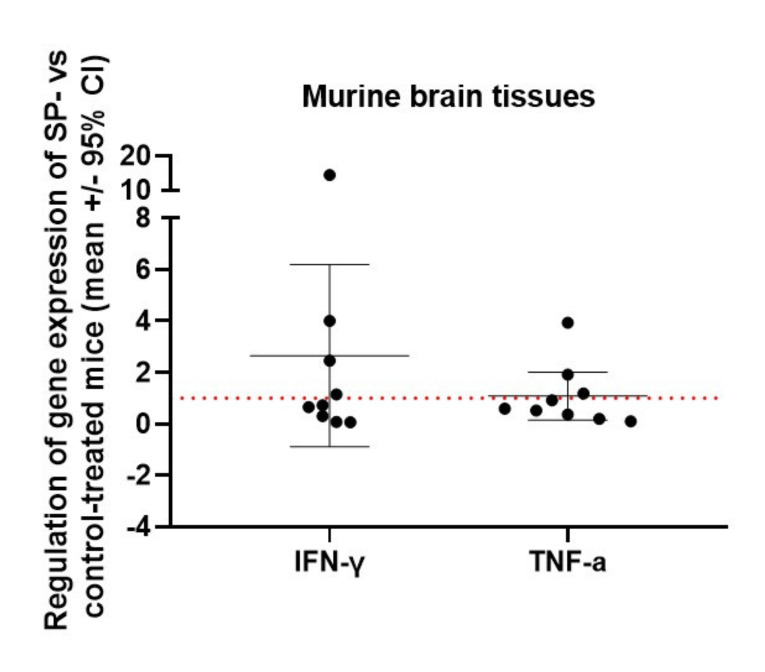
Effect of SP treatment on relative cytokine mRNA expression in murine brains of EAE-diseased mice (acute phase). X-fold difference in the relative expression of interferon gamma (INF-γ) and tumor necrosis factor alpha (TNF-α) (normalized to Rps18) in SP 30 mg/kg/day-treated mice compared to control mice. Control (*n* = 9, in triplicates), SP 30 mg/kg/day (*n* = 9, in triplicates) and 3 days of treatment. qRT-PCR. Abbreviations: SP: SP600125 and CI: confidence of interval. Statistic: one-sample *t*-test: n.s.

**Figure A4:**
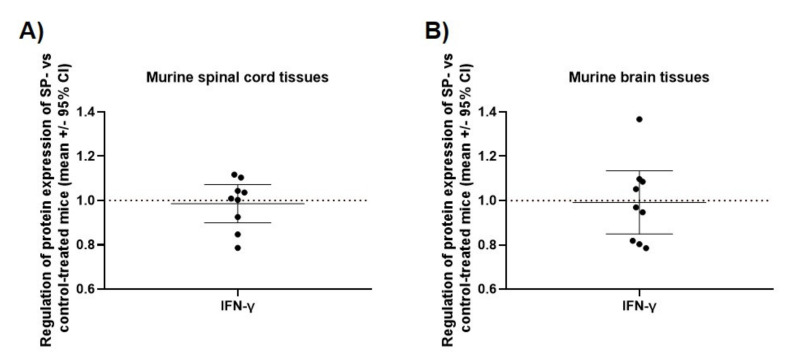
Effect of SP treatment on relative cytokine protein INF-γ expression in (**A**) murine spinal cords and in (**B**) murine brains of EAE-diseased mice (acute phase). X-fold difference in the relative expression of INF-γ in SP 30 mg/kg/day-treated mice compared to control mice. Control (*n* = 9, in triplicates), SP 30 mg/kg/day (*n* = 9, in triplicates) and 3 days of treatment. ELISA. Abbreviations: SP: SP600125 and CI: confidence of interval. Statistic: one-sample *t*-test: n.s.

In spinal cord, SP treatment (30 mg/kg/day) led to a 2.4-fold increased mRNA expression of IFN-γ and a 2.1-fold increase of TNF-α mRNA expression compared to control mice in the acute phase of MOG_35-55_ EAE (OSST (test value 1): *p* < 0.05; Figure 3A), whereas IL-4, IL-10 and IL-17 were not differentially regulated (OSST (test value 1): Each *p*-value > 0.05; Figure 3A). No significant different mRNA expression of INF-γ and TNF-α was observed in the brains of SP-treated acute MOG_35-55_ EAE-diseased mice compared to control mice (OSST (test value 1): *p* > 0.05, Figure A3). Spinal cord and brain protein expression of INF-γ also did not significantly differ between the two groups (OSST (test value 1): *p* > 0.05, Figure A4). In splenic-derived CD3+ T cells, no differential cytokine mRNA expression between SP (30 mg/kg/day) and control treatment was detected (OSST (test value 1): Each *p* > 0.05; Figure 3B). Clinical disease course and body weight of the mice used for these experiments are shown in Figure A1 and Figure A2.

### 3.4. JNK Inhibition Reduces Spinal Cord Cell-Infiltration during the Acute Phase of MOG_35-55_ EAE

**Figure 4:**
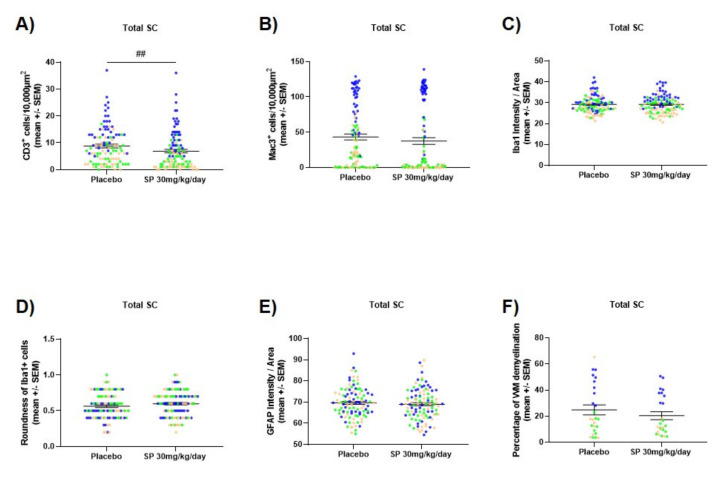
Effect of SP treatment on murine spinal cord (**A**) CD3+ cell infiltration, (**B**) Mac3+ cell infiltration, (**C**) Iba1 fluorescence intensity, (**D**) Iba1+ cell roundness, (**E**) GFAP fluorescence intensity and (**F**) white matter demyelination. Spinal cords were extracted from control or SP 30 mg/kg/day-treated MOG_35-55_ EAE-diseased mice (acute phase). Three segments of each spinal cord (cervical, thoracic and lumbar) were evaluated and pooled. For each segment, four different regions of interest were analyzed except for demyelination where the complete white matter was analyzed. Control (n = 9), SP 30 mg/kg/day (*n* = 9) and 3 days of treatment. (**A**–**B**) immunohistochemistry, (**C**–**E**) immunofluorescence and (**F**) luxol fast blue staining. Each dot represents a measurement. Orange dots show measurements of the cervical segment, green of the thoracic segment and blue of the lumbar segment of the spinal cord. Abbreviations: SC: Spinal cord, SEM: Standard error of the mean, SP: SP600125 and WM: White matter. Statistic: Mann–Whitney test, ## < 0.01.

**Figure A5:**
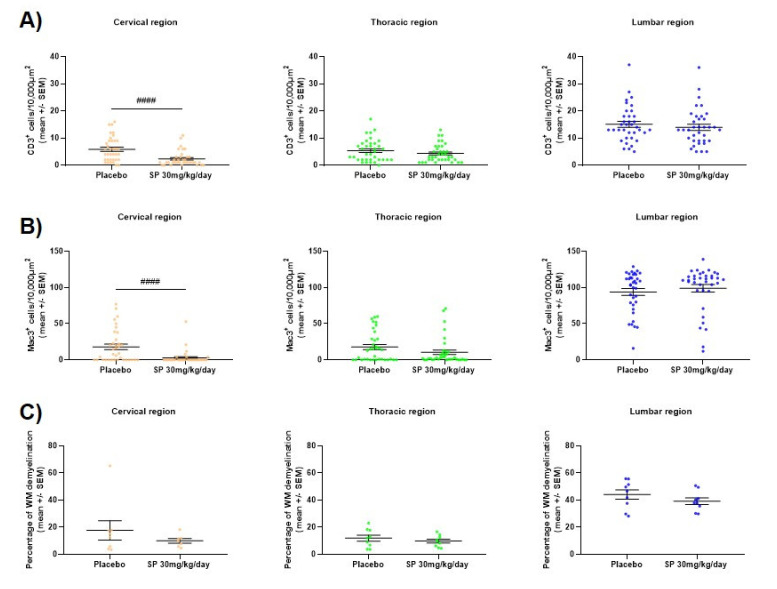
Effect of SP treatment on murine spinal cord (**A**) CD3+ cell infiltration, (**B**) Mac3+ cell infiltration and (**C**) demyelination. Spinal cords were extracted from control or SP 30 mg/kg/day-treated EAE-diseased mice (acute phase). Three segments of each spinal cord (cervical, thoracic and lumbar) were evaluated. For each segment, four different regions of interest were analyzed except for demyelination where the complete white matter was analyzed. Control (*n* = 9), SP 30 mg/kg/day (*n* = 9) and 3 days of treatment. (**A**–**B**) immunohistochemistry and (C) luxol fast blue staining. Each dot represents a measurement. Abbreviations: SC: spinal cord, SEM: standard error of the mean, SP: SP600125 and WM: White matter. Statistic: Mann–Whitney test, #### < 0.0001.

**Figure A6:**
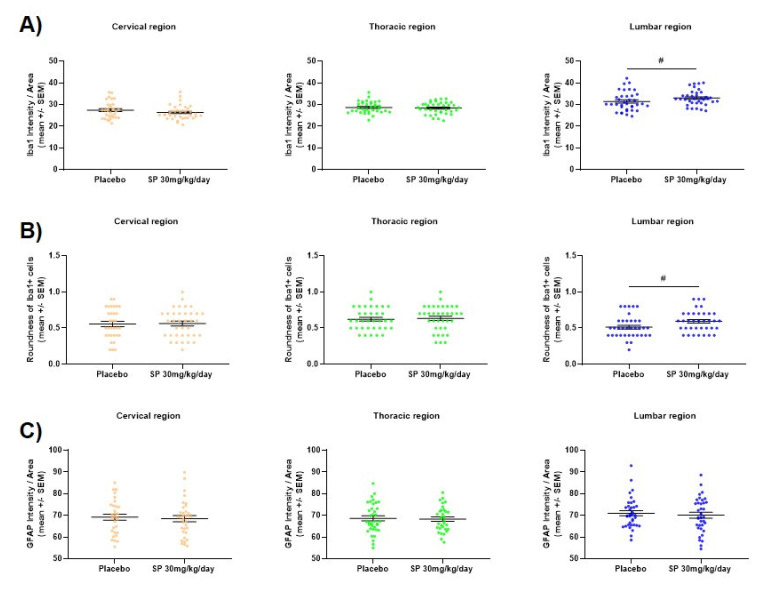
Effect of SP treatment on murine spinal cord (**A**) Iba1 fluorescence intensity, (**B**) Iba1+ cell roundness and (**C**) GFAP fluorescence intensity. Spinal cords were extracted from control or SP 30 mg/kg/day-treated EAE-diseased mice (acute phase). Three segments of each spinal cord (cervical, thoracic and lumbar) were evaluated. For each segment, four different regions of interest were analyzed. Control (*n* = 9), SP 30 mg/kg/day (*n* = 9) and 3 days of treatment. (**A**–**C**) immunohistochemistry. Each dot represents a measurement. Abbreviations: SC: Spinal cord, SEM: Standard error of the mean and SP: SP600125. Statistic: Mann–Whitney test, # < 0.05.

SP 30 mg/kg/day-treated acute MOG_35-55_ EAE-diseased mice showed a significant reduced spinal cord CD3+ T cell infiltration compared to control mice (MWT, *p* < 0.01, Figure 4A). This reduced CD3+ T cell infiltration is pronounced when looking at the cervical segment of the spinal cord (MWT, *p* < 0.0001, Figure A5A). A trend at a 90% level of significance of a reduced Mac3+ cell infiltration in the overall spinal cord was also observed (MWT, *p* = 0.07, Figure 4B) which was driven by the cervical segment (MWT, *p* < 0.0001, Figure A5B). Significant increase of Iba1 fluorescence intensity, as well as a significant increase in the roundness of the Iba1+ cells was noticed in the lumbar segment of the spinal cord (MWT, *p* < 0.05, Figure A6A–B) but no significance was observed when looking at the overall spinal cord (MWT, *p* > 0.05, Figure 4C–D). No significantly different GFAP fluorescence intensity was observed between the two groups, neither in all nor in a specific segment (MWT, *p* > 0.05, Figure 4E and Figure A6C). Furthermore, no significant difference in white matter demyelination was noticed between the two groups (MWT, *p* > 0.05, Figure 4F and Figure A5C).

### 3.5. JNK inhibition Exerts Anti-Apoptotic and Anti-Proliferative Effects in Murine and Human CD3+ T Cells

**Figure 5:**
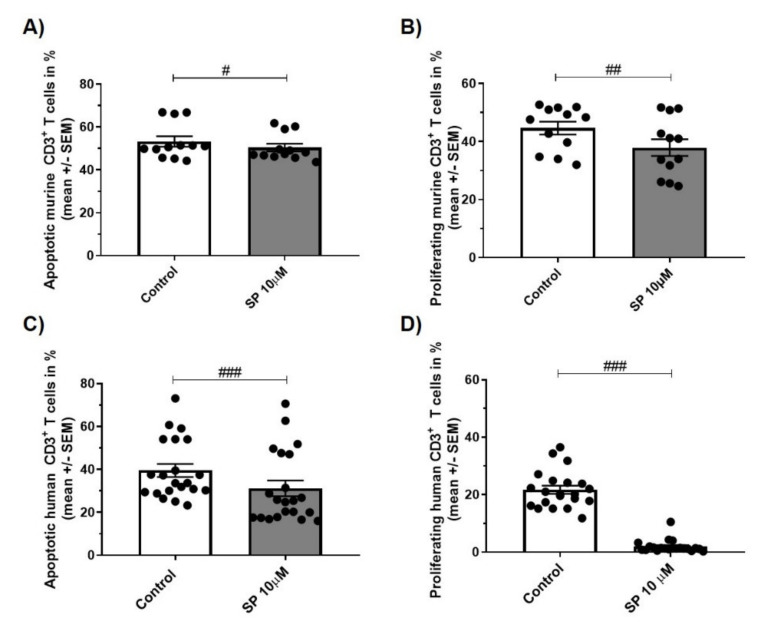
(**A**,**C**) apoptosis of CD3+ T cells of C57BL/6JRj mice (**A**, *n* = 4 experiments in triplicates, stimulus concanavalin A (conA) 1.5 µg/mL, 24 h) and healthy human controls (**C**, *n* = 7 experiments in triplicates, stimulus phytohemagglutinin (PHA) 0.5 µg/mL, 72 h). Incubation with control or SP 10 µM (Annexin V/PI, flow cytometry). (**B**,**D**) proliferation of CD3+ T cells of C57BL/6JRj mice (**B**, *n* = 4 experiments in triplicates, stimulus conA 1.5 µg/mL, 24 h) and healthy human controls (**D**, *n* = 7 experiments in triplicates, stimulus PHA 0.5 µg/mL, 72 h). Incubation with control or SP 10 µM (CFSE, flow cytometry). Abbreviations: SEM: Standard error of the mean and SP: SP600125. Statistic: Wilcoxon signed rank test # < 0.05, ## < 0.01 and ### < 0.001.

SP administration reduced apoptosis and proliferation of CD3+ T cells from C57BL/6 WT mice and human healthy donors in vitro: Incubation of murine CD3+ T cells with SP 10 µM resulted in a reduction of apoptosis by 5% compared to control (WSRST: *p* < 0.05; Figure 5A) and reduced proliferation by 15% (WSRST: *p* < 0.01; Figure 5B). Results in the human situation were more pronounced with a reduction of CD3+ T cell apoptosis by21% (WSRST: *p* < 0.001; Figure 5C) and decreased proliferation by 91% (WSRST: *p* < 0.001; Figure 5D). 

## 4. Discussion

**Figure 6:**
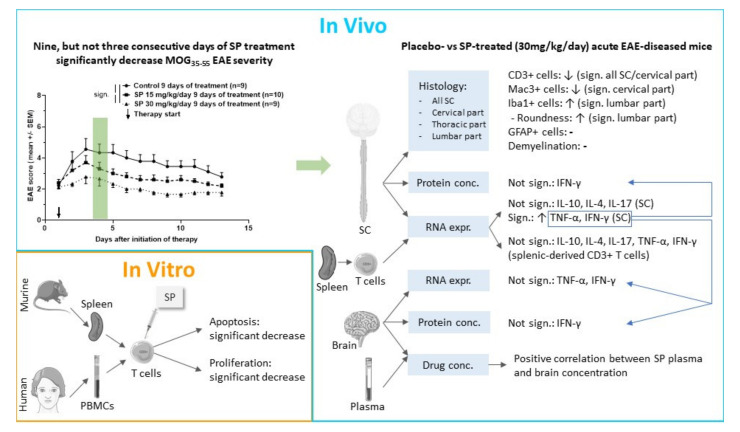
Graphical abstract summarizing key experiments and results of this study. Abbreviations: conc.: Concentration, EAE: Experimental autoimmune encephalomyelitis, expr.: Expression, sign.: Significant, SC: Spinal cord, SP: SP600125 and PBMCs: Peripheral blood mononuclear cells. This figure was created using Servier Medical Art templates licensed under a Creative Commons License (https://creativecommons.org/licenses/by/3.0/).

Using two different in vivo treatment regimens, our study demonstrated that pharmacological pan-JNK inhibition ameliorates MOG_35-55_ EAE disease course when administered after disease onset over nine consecutive days in a therapeutic setting. Effects of pan-JNK inhibition appear to be mediated by inhibition of CD3+ T cell proliferation and apoptosis in the peripheral immune compartment and by regulation of cytokine expression and cell infiltration in the CNS (Figure 6). 

Clinical effects were detected in MOG_35-55_ EAE experiments using SP, a pharmacological pan-JNK inhibitor, given over nine consecutive days after disease onset. These findings contradict results of studies with JNK isoform-specific knockout models, which failed to demonstrate a significant impact on MOG_35-55_ EAE disease course [6]. Considering that JNK1 and JNK2 have important, but overlapping functions [6], clinical effects of pharmacological pan-JNK inhibition with similar specificity for these two isoforms [17] may explain these results, because reduced activity of one isoform cannot be compensated by increased activity of the other. Taking into account that a *Jnk-1*/*Jnk-2* double knockout has a lethal phenotype [21], drug safety of our pharmacological approach has to be addressed carefully. In this regard, our treatment regimen during either three or nine days of SP administration demonstrated no clinical signs of SP side effects like a pronounced reduction of body weight, altered animal behavior or even death. Regarding body weight as one parameter of drug toxicity [19], multivariate linear regression analysis demonstrated that no MOG_35-55_ EAE score-independent negative effect of SP treatment on body weight was present. Nevertheless, in addition to longer drug administration and higher dosages, other parameters of drug toxicity like drug serum and tissue concentration, hematological, liver and kidney parameters and daily food consumption should be investigated in the future to accurately study the feasibility of a pharmacological pan-JNK inhibition in the light of drug safety issues. 

Plasma and brain tissue concentrations of SP were measured 24 h after the third oral gavage. Preliminary results demonstrated a strong positive correlation (Pearson coefficient 0.97) between plasma and brain SP concentration, indicating that SP crosses the blood-brain barrier. Concentrations in the brain were approximately 2.6-fold higher than in the plasma, which might point to a favorable CNS biodistribution of SP supporting the treatment efficacy in CNS disease. 

Mechanistically, our study investigated cytokine regulation by SP during peak of MOG_35-55_ EAE disease in the peripheral immune (splenic-derived CD3+ T cells) and CNS compartment (spinal cord and brain). mRNA expression of Th1-derived cytokines (INF-γ, TNF-α), Th2-derived cytokines (IL-4), Th17-derived cytokines (IL-17) and regulatory cytokines (IL-10) were analyzed both in splenic-derived CD3+ T cells and in the spinal cord of acute MOG_35-55_ EAE-diseased mice [22]. We observed an increased mRNA expression of INF-γ and TNF-α in the spinal cord of SP 30 mg/kg/day-treated acute MOG_35-55_ EAE-diseased mice compared to control, whereas peripheral cytokine mRNA expression measured in splenic-derived CD3+ T cells was not significantly regulated by SP. TNF-α and INF-γ are both known to be secreted by different peripheral cells including T cells [23,24]. However, CNS-resident cells have also been shown to produce these two types of cytokines [25,26] suggesting that these cell types could be responsible for the observed increased mRNA expression of TNF-α and INF-γ in the spinal cord. In contrast, spinal cord protein expression of INF-γ did not significantly differ between treatment groups. However, this result does not contradict the previous one knowing that mRNA and protein concentration do not necessarily correlate [27]. Moreover brain mRNA and protein expression of INF-γ as well as brain mRNA of TNF-α were not affected by SP treatment, which is not surprising knowing that the EAE driven inflammation mainly affects the spinal cord [28]. TNF-α protein expression was also analyzed both in the spinal cord and brain of mice, however obtained values were below the detection range of the ELISA kit (8 pg/ml) prohibiting further analysis. Increased expression of INF-γ and reduced EAE disease severity has to be discussed in detail, as INF-γ was first described to exacerbate EAE and MS [29]. However, there is increasing evidence that INF-γ has beneficial effects on EAE, e.g., mice deficient for INF-γ or INF-γ receptor or mice treated with a neutralizing INF-γ monoclonal antibody have a more severe EAE disease course [30]. These contradictory findings of INF-γ can be explained using a dynamic model of INF-γ function under consideration of different EAE disease phases. More precisely, it has been proposed that INF-γ promotes pathogenesis during the initiation phase, whereas it suppresses the disease during the acute phase [31,32]. Translated to our work, the finding of increased INF-γ expression in spinal cord during the acute phase of MOG_35-55_ EAE might represent one molecular mechanism, which finally leads to a less severe disease course in SP-treated mice. In addition to INF-γ expression, TNF-α mRNA expression was upregulated in the spinal cord during the acute phase in SP 30 mg/kg/day-treated MOG_35-55_ EAE-diseased mice. The role of TNF-α in both EAE and MS is a matter of a controversial discussion. On one side, TNF inhibition has beneficial effects in EAE [33] and TNF receptor I deficiency leads to delayed EAE onset [34]. On the other side, results of a clinical trial using TNF inhibitor in MS patients were negative as MS disease was even worse in the treatment arm [35]. It has been demonstrated that mice lacking TNF show severe neurological impairment with extensive demyelination [36], and mice lacking the TNF receptor II have an increased disease severity [37]. Another study demonstrated the importance of differentiating the soluble and the membrane form of TNF with a detrimental role of soluble TNF and a beneficial role of the transmembrane TNF in EAE. In detail, they showed that soluble TNF enhances the production of pro-inflammatory mediators, whereas transmembrane TNF diminishes their production in CNS tissues at the peak of EAE disease [38]. In our study, the observed clinical effect of SP is associated with an increased TNF-α expression in the spinal cord during the acute phase of EAE disease, suggesting that increased TNF-α expression is associated with beneficial effects of SP treatment. However, a limitation of our study is that we are not able to differentiate between membrane-bound and soluble form of TNF by only analyzing the gene expression [25].

Several hallmarks such as T cell and macrophage CNS infiltration, demyelination or astrocyte and microglia activation characterize the EAE [39]. We observed a significant reduction of CD3+ T cell infiltration in the whole spinal cord during the peak of the disease. In more details, stronger significant reduction of CD3+ T cell infiltration was observed when looking at the cervical part of the spinal cord. Despite it being still a matter of debate, various evidence points towards a role of JNK on T cell activation/function [40,41,42,43,44]. We showed in vitro that JNK inhibition influences murine CD3+ T cell proliferation and apoptosis, supporting our histological findings. There is sparse and inconclusive evidence for effects of JNK inhibition on proliferation. Indeed, some demonstrated that SP reduces T cell proliferation [17], whereas others observed no effect [45]. Regarding apoptosis, an influence of the JNK pathway on pro- and anti-apoptotic mechanisms depending on cell type and stimulus is described [44]. Hypothetical, the functional effects of SP can be explained by the fact that T cell apoptosis is reduced when anergy, expressed as lowest proliferation response, is maximal [46]. Anergic T cells were shown to be defective in JNK signaling [47] and hindered JUN activation due to inhibited autophagy [48]. Murine in vitro experiments were translated to the human situation, confirming that in vitro effects of a pan-JNK inhibition were comparable between murine and human CD3+ T cells. This gives experimental evidence that a pan-JNK inhibition might also be a potential treatment strategy in human MS disease. 

Reduced CD3+ T cell infiltration was accompanied by a decreased macrophage, defined as Mac3+ cell, infiltration of the cervical part of the spinal cord. A beneficial effect of JNK inhibition on macrophage infiltration has already been reported in other diseases. Indeed, reduced macrophage infiltration into the colon has been observed in the dextran sulphate sodium (DSS)-induced colitis, an animal model of inflammatory bowel diseases, when treated with SP [49]. In addition, it has been demonstrated that activation of the JNK pathway was essential for the development and the survival of macrophages [50]. Moreover, the effect of JNK inhibition on both GFAP and Iba1 fluorescence intensity was analyzed. GFAP is commonly used to determine activated astrocytes [51,52], whereas Iba1 is a marker for both macrophages and microglia [53]. Iba1 fluorescence intensity as well as morphological analysis have been used to determine macrophage/microglia reactivity [54]. In our study, a significant increase of Iba1 fluorescence intensity, as well as a significant increase in the roundness of the Iba1+ cell was noticed in the lumbar segment of the spinal cord, which hints at an increased activity of microglia. Our findings are in line with previous data demonstrating that JNK inhibition with SP changes inflammatory metabolic activity of microglia after external pro-inflammatory stimulus with lipopolysaccharide in mice [55]. 

Despite effects on immune cell populations, we did not observe a statistically significant difference in white matter demyelination between control and SP 30 mg/kg/day-treated acute MOG_35-55_ EAE-diseased mice. Nevertheless, the white matter demyelination was lower in SP-treated mice at peak of disease (mean control mice: 24.8% (SEM 3.78); mean SP-treated mice: 20.5% (SEM 3.07)), also reflecting reduced clinical MOG_35-55_ EAE severity in the SP treated animals. Indeed, c-Jun, a downstream target of JNK, has previously been shown to negatively regulate myelination by inhibiting different myelin genes and by favoring an immature state of the myelinating cells [56]. 

In our study, the effect of a pan-JNK inhibitor was analyzed and consequently no distinction between the different isoforms was made. However, it is worthwhile to mention that several studies point toward an important role of JNK3 inhibition in neuroprotection. Indeed, it has been observed that mice with a neural-specific *Jnk3* deletion are more resistant to glutamate excitotoxicity [57], which has been shown to be deleterious in the EAE [58,59]. Furthermore, it has been shown that disruption of the *Jnk3* gene render mice less sensitive to brain injury after cerebral ischemia and that it prevents the release of the cytochrome c from the mitochondria after oxygen-glucose deprivation [60]. Interestingly, it has also been shown that both TNF- and TNF-related apoptosis-inducing ligand (TRAIL)-induced death of mature adult human oligodendrocytes are mediated by JNK3 [61,62]. Consequently, further investigations are warranted in order to define the therapeutic value of JNK inhibitors in MS better. 

Findings of our experimental study should promote further investigations in the future taking into account clinical effects, biological mechanisms of action as well as drug tolerability of pan-JNK inhibition.
